# Social media addiction and cyberbullying sensibility in team sports athletes: the mediating role of digital literacy and the moderating role of difficulties in emotion regulation

**DOI:** 10.3389/fpsyg.2026.1890888

**Published:** 2026-06-23

**Authors:** Hasan Buğra Ekinci, Mehmet Cüneyt Birkok, Zülbiye Kaçay, Özkan Işık, Laurentiu Gabriel Talaghir, Daniel Mădălin Coja, Anamaria Berdila, Bogdan Sorin Olaru

**Affiliations:** 1 Faculty of Sport Sciences, Erzincan Binali Yildirim University, Erzincan, Türkiye; 2 Faculty of Human and Public Sciences, Sakarya University, Sakarya, Türkiye; 3 Faculty of Sports Sciences, Çanakkale Onsekiz Mart University, Çanakkale, Türkiye; 4 Faculty of Sports Sciences, Balikesir University, Balikesir, Türkiye; 5 Faculty of Physical Education and Sport, Dunarea de Jos University of Galati, Galati, Romania

**Keywords:** cyberbullying sensibility, digital literacy, emotion regulation, social media addiction, team sports athletes

## Abstract

**Purpose:**

This study examined the relationship between social media addiction and cyberbullying sensibility among team sports athletes. In addition, the mediating role of digital literacy and the moderating role of difficulties in emotion regulation were investigated within this relationship.

**Methodology:**

The study included 265 licensed team sports athletes from Erzincan, Türkiye. Data were collected using measures of social media addiction, cyberbullying sensibility, digital literacy, and difficulties in emotion regulation. The construct validity of the scales was assessed through confirmatory factor analysis (CFA). Mediation, moderation, and moderated mediation analyses were conducted using PROCESS Macro Models 4 and 14 with bootstrap-based regression procedures.

**Result:**

The findings showed that social media addiction was significantly associated with digital literacy, and digital literacy showed a significant positive relationship with cyberbullying sensibility. The indirect relationship between social media addiction and cyberbullying sensibility through digital literacy was statistically significant. In addition, this indirect association was more pronounced among athletes with lower difficulties in emotion regulation, whereas it was not significant among those with higher difficulties in emotion regulation. These findings support a moderated mediation model based on conditional associations.

**Conclusion:**

The findings suggest that digital literacy plays a mediating role in the relationship between social media addiction and cyberbullying sensibility among team sports athletes. Furthermore, difficulties in emotion regulation appear to influence the strength of this indirect relationship. The study highlights the importance of considering both digital competencies and emotional processes in understanding athletes' experiences in digital environments.

## Introduction

In the digital age, social media has become a central component of interpersonal communication and information exchange, particularly among young individuals and athletes (Wu and Kuang, 2021; Valkenburg and Peter, 2011). Although these platforms facilitate communication, self-presentation, and access to information, they also expose users to various psychosocial risks. Excessive engagement with social media may lead individuals to lose control over their online behaviors and increase their vulnerability to negative digital experiences, including cyberbullying (Kowalski et al., 2014). Moreover, the characteristics of online communication environments may reduce sensitivity to the emotional consequences of harmful interactions and contribute to the normalization of aggressive online behaviors.

Athletes constitute one of the groups most visibly exposed to such digital risks. Due to the public nature of their performances and their accessibility through social media platforms, athletes are frequently subjected to negative comments, insults, and threatening content. Recent incidents involving collegiate athletes, such as Angel Reese, Gabbie Marshall, and RJ Luis Jr., have highlighted the growing visibility of online harassment directed toward athletes following competitive performances (Boone, 2024; Shapiro, 2025). These experiences may negatively affect athletes' psychological wellbeing, emotional functioning, and performance. Similarly, Yücetürk and Agin (2022) emphasized that cyberbullying may have long-term psychological and social consequences for athletes. In this respect, understanding the factors associated with cyberbullying sensibility among athletes has become increasingly important.

Cyberbullying refers to intentional and repetitive harmful behaviors carried out through digital technologies and online communication platforms. Exposure to such experiences may create a perception of psychological threat and increase individuals' awareness of potential risks in digital environments. Cyberbullying sensibility is conceptualized in this study as a proactive cognitive construct that integrates awareness of harmful online behaviors with the readiness to employ protective strategies in digital environments. This construct is distinct from behavioral forms of cyberbullying involvement. Specifically, cyberbullying victimization refers to being exposed to harmful or aggressive online behaviors, whereas cyberbullying perpetration refers to the act of engaging in such behaviors. In contrast, cyberbullying sensibility represents a preventive psychological orientation characterized by the ability to recognize potential digital risks and respond to them before they escalate into harmful experiences (Tanrikulu et al., 2013). For athletes, who are highly visible in online environments, this sensitivity may be particularly important in maintaining psychological resilience and managing online interactions effectively.

Within this context, digital literacy and emotion regulation represent two important psychological and cognitive resources associated with coping in digital environments. Digital literacy refers to the ability to access, evaluate, and use digital information effectively and safely (Hobbs, 2010, 2011). Recent research in sport-related contexts has also suggested that digital behavioral patterns may influence sport-related psychological processes through cognitive mechanisms such as self-regulation and cognitive flexibility (Özbek et al., 2026). Individuals with higher digital literacy may be more capable of identifying harmful online content, recognizing cyberbullying-related risks, and responding more consciously to digital interactions. Therefore, digital literacy may play a protective role in understanding the relationship between social media addiction and cyberbullying sensibility.

Emotion regulation, on the other hand, refers to the processes through which individuals monitor, evaluate, and regulate their emotional responses, particularly under stressful or negative conditions (Gross, 1998). Difficulties in emotion regulation may influence how individuals perceive and respond to harmful digital experiences such as cyberbullying (Thompson, 1994). Athletes with lower difficulties in emotion regulation may respond more adaptively to stressful online interactions, whereas those with greater difficulties may become more vulnerable to the psychological effects of digital threats.

Although previous studies have separately examined social media use, cyberbullying, digital literacy, and emotion regulation, limited research has investigated these variables together within an integrated framework involving athletes. Therefore, the present study aims to examine the relationship between social media addiction and cyberbullying sensibility among team sports athletes. In addition, the mediating role of digital literacy and the moderating role of difficulties in emotion regulation are examined within this relationship.

By addressing digital literacy and emotion regulation together, this study contributes to a better understanding of the cognitive and emotional processes associated with athletes' experiences in digital environments. In this regard, the proposed model offers a comprehensive perspective on the psychological mechanisms underlying cyberbullying sensibility in team sports athletes.

## Literature review

### Theoretical framework

The theoretical basis of this study is grounded in Social Cognitive Theory (Bandura, 1986) and Cognitive Appraisal Theory (Lazarus and Folkman, 1984), both of which provide a useful framework for understanding individuals' emotional and cognitive responses in digital environments.

Social Cognitive Theory proposes that behavior develops through reciprocal interactions between environmental influences, personal characteristics, and prior experiences. Within digital environments, individuals' behaviors on social media may be shaped by exposure to online content, personal competencies such as digital literacy, and psychological characteristics related to emotional functioning. In this context, excessive engagement with social media may increase individuals' exposure to online risks and make them more vulnerable to negative digital experiences, including cyberbullying.

Cognitive Appraisal Theory focuses on how individuals evaluate and respond to potentially stressful situations. According to this perspective, individuals cognitively appraise events as threatening, harmful, or manageable, and these evaluations influence their emotional and behavioral responses. In the context of cyberbullying, athletes' perceptions of digital threats and their ability to regulate emotional reactions may influence their level of cyberbullying sensibility.

Based on these theoretical perspectives, the present study assumes that social media addiction may be associated with cyberbullying sensibility among athletes, and that this relationship may be explained through digital literacy and conditioned by difficulties in emotion regulation. Accordingly, the proposed model provides an integrated framework for understanding cognitive and emotional processes associated with digital experiences among team sports athletes.

### Background

Social media has transformed the ways individuals access, share, and interact with information. Particularly among young individuals and athletes, digital platforms function not only as communication environments but also as spaces for social interaction and self-presentation. However, increased engagement with social media may also increase exposure to harmful digital experiences such as cyberbullying (Kowalski et al., 2014).

The relationship between social media addiction and cyberbullying sensibility may be partially explained through digital literacy. Digital literacy refers to individuals' ability to critically evaluate digital content, access reliable information, and engage safely in online environments (Eshet-Alkalai, 2004). Individuals with higher levels of digital literacy may be more capable of recognizing harmful online behaviors and identifying potential cyberbullying risks. Recent research in sport-related contexts has also suggested that digital behavioral patterns may influence psychological processes through cognitive mechanisms such as self-regulation and cognitive flexibility (Oliveira et al., 2025). Although digital literacy is frequently conceptualized as a protective competence, it may also develop through sustained engagement with digital environments (Livingstone, 2014). Individuals who are regularly immersed in social media platforms are repeatedly exposed to diverse forms of digital communication, online information systems, and technology-mediated interactions. Such exposure may enhance familiarity with digital tools and contribute to the gradual development of digital literacy skills through experiential learning processes in complex digital environments (Livingstone, 2014).

Within the context of athletes, social media platforms are not merely used for entertainment purposes but also serve as essential tools for communication, self-presentation, performance branding, and managing professional visibility (Kavanagh et al., 2023). Recent evidence further suggests that digital competencies among university athletes are closely embedded in their daily online practices and broader psychological functioning (Tek and Özsari, 2025).

From this perspective, intensive engagement with social media environments may be associated with higher levels of digital literacy due to repeated exposure to digital communication practices and information management processes. Accordingly, social media addiction may coexist with digital literacy, and digital literacy may function as a cognitive mechanism through which social media addiction is associated with cyberbullying sensibility.

At the same time, increasing interaction within digital environments requires individuals to develop both technical competencies and ethical awareness regarding online communication. However, previous studies have shown that some individuals may perceive cyberbullying behaviors as a normalized aspect of digital interaction (Aldao et al., 2010). This normalization may reduce awareness of harmful online experiences and weaken sensitivity toward cyberbullying. Therefore, digital literacy may play an important role in understanding cyberbullying sensibility in athletes.

H1: Social media addiction is positively associated with digital literacy, which in turn is positively associated with cyberbullying sensibility. Accordingly, digital literacy mediates the relationship between social media addiction and cyberbullying sensibility.

Emotion regulation refers to the processes through which individuals recognize, monitor, and manage their emotional reactions under stressful conditions (Gross, 1998). Difficulties in emotion regulation may influence how individuals respond to harmful online experiences such as cyberbullying. Individuals with lower difficulties in emotion regulation may interpret negative online experiences more adaptively and may therefore be less psychologically vulnerable to digital threats (Thompson, 1994; Christensen et al., 2015).

Accordingly, difficulties in emotion regulation may influence the relationship between digital literacy and cyberbullying sensibility. In other words, the relationship between digital literacy and cyberbullying sensibility may differ depending on individuals' levels of emotion regulation difficulties.

H2: Difficulties in emotion regulation moderate the relationship between digital literacy and cyberbullying sensibility.

Furthermore, the indirect relationship between social media addiction and cyberbullying sensibility through digital literacy may vary according to individuals' difficulties in emotion regulation.

H3: Difficulties in emotion regulation moderate the indirect relationship between social media addiction and cyberbullying sensibility through digital literacy.

## Methodology

### Research model

This study aimed to examine the relationship between social media addiction and cyberbullying sensibility among team sports athletes. In addition, the mediating role of digital literacy and the moderating role of difficulties in emotion regulation were investigated within this relationship.

**Figure 1:**
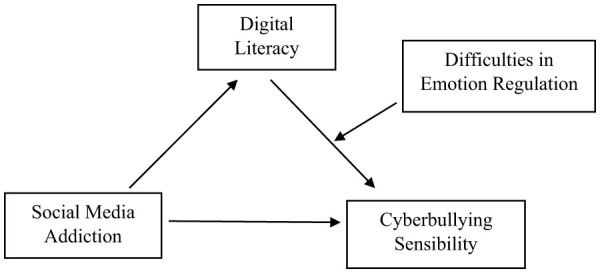
Research model.

The study was conducted using a relational survey model, which is commonly employed in quantitative research to examine relationships among variables (Aldao et al., 2010; Christensen et al., 2015; Karasar, 2023). In line with the proposed theoretical framework, a moderated mediation model was developed to test the direct and indirect relationships between the study variables. The theoretical model of the study is presented in Figure 1.

### Research group

The study group was determined using the convenience sampling method, which involves selecting participants who are easily accessible to the researchers (Cohen et al., 2000). *A priori* power analysis was conducted using the G^*^Power program to determine the required sample size (Richard et al., 2003). Based on a statistical power level of 0.80, an alpha level of 0.05, and an effect size corresponding to a correlation coefficient of 0.20, the minimum required sample size was calculated as 193 participants. However, increasing sample size may improve the reliability of findings and reduce estimation errors (Faul et al., 2009). Accordingly, the study was conducted with 265 participants.

The sample consisted of 265 licensed team sports athletes actively participating in sports clubs in Erzincan, Türkiye. Of the participants, 135 were male and 130 were female, with a mean age of 21.4 years. Regarding sports experience, 16.2% of the participants had been actively involved in sports for 0–2 years, 41.9% for 3–5 years, and 42.1% for 6 years or more.

### Data collection tools

#### Social media addiction scale

Social media addiction was assessed using the Bergen Social Media Addiction Scale (BSMAS) (Andreassen et al., 2016). The scale consists of six items rated on a 5-point Likert scale ranging from 1 (“very rarely”) to 5 (“very often”). Higher scores indicate higher levels of social media addiction. The Turkish adaptation of the scale was conducted by Demirci (2019), who reported satisfactory reliability and validity findings (Demirci, 2019).

In the present study, the Cronbach's alpha coefficient of the scale was calculated as 0.851. Confirmatory factor analysis indicated excellent model fit indices (χ^2^/df = 0.82, CFI = 1.000, TLI = 1.004, RMSEA = 0.000, SRMR = 0.017), suggesting that the one-factor structure of the scale was supported (Hu and Bentler, 1999). Modification index analysis indicated a significant covariance between error terms e1 and e2, and this covariance was included in the model without altering the theoretical structure of the scale. In addition, the AVE and CR values were calculated as 0.498 and 0.89, respectively. Although the AVE value was slightly below the recommended threshold of 0.50, the CR value exceeded the acceptable criterion of 0.70, supporting the construct validity of the scale (Fornell and Larcker, 1981).

#### Cyberbullying sensibility scale

Cyberbullying sensibility was measured using the Cyberbullying Sensibility Scale developed by Tanrikulu et al. (2013). The scale was finalized after two pilot applications and consists of 13 items under a single-factor structure. The original study reported that the scale explained 46.66% of the total variance, and confirmatory factor analysis supported the factorial structure (χ^2^/df = 3.220, RMSEA = 0.082). Items are scored on a three-point scale: “Yes” (3 points), “Sometimes” (2 points), and “No” (1 point). Higher scores indicate higher levels of cyberbullying sensibility. The original study reported Cronbach's alpha coefficient of 0.79 (Tanrikulu et al., 2013).

In the present study, the Cronbach's alpha coefficient for the scale was calculated as 0.806. Confirmatory factor analysis demonstrated acceptable model fit indices [χ^2^/df = 2.193, CFI = 0.925, TLI = 0.907, RMSEA = 0.067 (90% CI = 0.052–0.082), SRMR = 0.054], indicating that the measurement model showed an acceptable fit to the data (Hu and Bentler, 1999). Modification indices suggested covariance relationships between e1 ↔ e2 and e10 ↔ e12, which were incorporated into the model without altering the theoretical structure of the scale. In addition, the AVE and CR values were calculated as 0.51 and 0.88, respectively, supporting the construct validity of the scale.

#### Digital literacy scale

Participants' digital literacy levels were assessed using the Digital Literacy Scale developed by Ng (2012) and adapted into Turkish by Ustundag et al. (2017). The scale consists of 10 items under a single-factor structure and explains approximately 40% of the total variance. Items are rated on a five-point Likert scale ranging from 1 (“Strongly disagree”) to 5 (“Strongly agree”). The scale does not contain reverse-coded items.

The Turkish adaptation study reported a Cronbach's alpha coefficient of 0.86, whereas the internal consistency coefficient in the current study was calculated as 0.920. Confirmatory factor analysis indicated acceptable model fit indices [χ^2^/df = 2.478, CFI = 0.970, TLI = 0.957, RMSEA = 0.075 (90% CI = 0.054–0.096), SRMR = 0.033], supporting the adequacy of the measurement model (Hu and Bentler, 1999). Furthermore, AVE and CR values were found to be 0.53 and 0.91, respectively, indicating satisfactory construct validity.

#### Difficulty in emotion regulation scale

Difficulties in emotion regulation were assessed using the short form of the Difficulties in Emotion Regulation Scale (DERS-16). The original scale was developed by Gratz and Roemer (Gratz and Roemer, 2004), and a shorter 16-item version was later developed by Bjureberg et al. (2016). The Turkish adaptation of the scale was conducted by (Yigit and Guzey Yigit 2019).

The scale consists of 16 items rated on a five-point Likert scale ranging from 1 (“rarely”) to 5 (“almost always”). Higher scores indicate greater difficulties in emotion regulation. In the present study, analyses were conducted using the total score, and subdimensions were not analyzed separately.

The Cronbach's alpha coefficient for the scale was calculated as 0.710. Confirmatory factor analysis demonstrated acceptable model fit indices [χ^2^/df = 1.973, CFI = 0.932, TLI = 0.921, RMSEA = 0.061 (90% CI = 0.048–0.073), SRMR = 0.041, GFI = 0.907], indicating good model fit (Hu and Bentler, 1999). Modification indices suggested covariance relationships between e1 ↔ e2, e3 ↔ e5, and e5 ↔ e6. These modifications improved model fit without affecting the theoretical integrity of the scale. In addition, the AVE and CR values were found to be 0.52 and 0.89, respectively, supporting the construct validity of the measure.

#### Process

This study examined the relationship between social media addiction and cyberbullying sensibility, together with the potential underlying psychological mechanisms. First, the mediating role of digital literacy in the relationship between social media addiction and cyberbullying sensibility was tested. Second, the moderating role of difficulties in emotion regulation in the relationship between digital literacy and cyberbullying sensibility was examined. Finally, a moderated mediation model was tested to determine whether the indirect relationship between social media addiction and cyberbullying sensibility through digital literacy varied according to the level of difficulties in emotion regulation.

#### Data analysis

The research data were analyzed using SPSS 22.0 software IBM SPSS Statistics 22.0 (IBM Corporation, Armonk, New York, USA). Prior to the main analyses, missing data, outliers, and assumptions of normality were examined using skewness, kurtosis, *Z* scores, and Mahalanobis distance values. The findings indicated that the data were normally distributed and that no multicollinearity problem was present.

Descriptive statistics and Pearson product–moment correlation analyses were conducted to examine the distributions of the study variables and the bivariate relationships among social media addiction, cyberbullying sensibility, digital literacy, and difficulties in emotion regulation.

The factorial structures of the measurement tools were evaluated through confirmatory factor analysis (CFA) using AMOS 26 IBM SPSS AMOS 26 (IBM Corporation, Armonk, New York, USA). The study hypotheses were tested using PROCESS Macro Models 4 and 14 developed by (Hayes, 2022). Bootstrap-based regression analyses were conducted using 5,000 resamples and a 95% confidence interval. In line with previous recommendations, indirect effects were considered statistically significant when the confidence interval did not include zero (Hayes et al., 2017; Preacher and Hayes, 2008).

#### Common method bias test

Harman's one-factor test was applied to assess the potential effect of common method bias (CMB). In this context, all measurement items were included in the analysis using Principal Component Analysis. According to the analysis results, a multi-factor structure with an eigenvalue greater than 1 was obtained, and a total of eleven factors emerged. This indicates that the data cannot be explained by a unidimensional structure and instead possesses a multi-factor structure. When considering the single-factor solution, it was determined that the first factor explained 27.215% of the total variance. The literature states that for common method variance to be considered problematic, a single factor must explain more than 50% of the total variance (Eichhorn, 2014). In this context, the obtained value falls below the relevant threshold, indicating that common method bias does not pose a serious threat. Additionally, the highest correlation coefficient among the variables was found to be *r* = −0.863. This value falls below the high correlation threshold (*r* > 0.90) considered problematic in the literature as an indicator of common method variance (Podsakoff et al., 2024). This finding also supports the conclusion that there is no excessive overlap among the variables and that the risk of common-method bias remains limited. Overall, when both the variance results from Harman's one-factor test and the maximum correlation value among the variables are evaluated together, it is concluded that common-method bias does not pose a significant problem in the research data.

## Results

### Correlation analysis and descriptive statistics

Descriptive statistics, correlation coefficients, and reliability values for social media addiction, cyberbullying sensibility, digital literacy, and difficulties in emotion regulation are presented in Table 1.

**Table 1:** Correlations between variables and descriptive analyses.

Variables	SMA	CBS	DL	DERS	X	Sd	Skewness	Kurtosis	Cronbach
SMA	1	0.047	0.255^**^	−0.194^**^	17.71	5.31	−0.037	0.104	0.851
CBS		1	0.209^**^	−0.200^**^	30.84	5.21	−0.121	−0.333	0.806
DL			1	−0.863^**^	35.46	7.98	−0.549	0.905	0.920
DERS				1	42.39	10.8	0.398	0.294	0.710

^*^p < 0.05, ^**^p < 0.01.

Table 1 presents descriptive statistics (*X* = mean, SD = standard deviation), normality indicators (skewness and kurtosis), Pearson correlation coefficients, and Cronbach's alpha reliability values for the main study variables: social media addiction (SMA), cyberbullying sensibility (CBS), digital literacy (DL), and emotion regulation (DERS).

Digital literacy and emotion regulation difficulties were significantly and negatively correlated (*r* = −0.863, *p* < 0.01), indicating a strong inverse association. Additionally, SMA was positively associated with DL (*r* =0.255, *p* < 0.01) and negatively associated with DERS (*r* = −0.194, *p* < 0.01). CBS also showed significant correlations with both DL (*r* =0.209, *p* < 0.01) and DERS (*r* = −0.200, *p* < 0.01).

All scales demonstrated acceptable internal consistency (Cronbach's α >0.70). The reliability coefficients were DL (α =0.920), SMA (α =0.851), CBS (α =0.806), and DERS (α =0.710).

According to Table 2, the results of Model 1 (H1) indicate that social media addiction significantly and positively predicts digital literacy [*b* =0.383, *p* < 0.001, 95% CI (0.207, 0.560)]. However, the direct relationship between social media addiction and cyberbullying sensibility was not statistically significant [*b* = −0.006, *p* =0.914, 95% CI (−0.127, 0.114)].

**Table 2:** Testing digital literacy on cyberbullying sensibility.

Predictors model 1 (H1)	DL (M)				CBS (Y)			
	*b*	*t*	SE	95% CI	*b*	*t*	SE	95% CI
SMA (X)	0.383^***^	4.27	0.089	0.206/0.559	−0.006	−0.108	0.061	−0.127/0.114
DL (M)	-		-	-	0.137^**^	3.37	0.040	0.057/0.217
*R* ^2^	0.043							
Indirect impact	SMA → DL → CBS *b* = 0.053^*^, 95% CI [0.0126, 0.1034]							

Bootstrap sample size = 5,000. LL, low limit; CI, confidence interval; UL, upper limit. ^***^p < 0.001, ^**^p < 0.01, ^*^p < 0.05.

Digital literacy was found to significantly predict cyberbullying sensibility [*b* = 0.138, *p* = 0.001, 95% CI (0.057, 0.218)]. In addition, the indirect relationship between social media addiction and cyberbullying sensibility through digital literacy was statistically significant [*b* = 0.053, 95% CI (0.013, 0.103)]. These findings suggest that digital literacy mediates the relationship between social media addiction and cyberbullying sensibility, supporting H1.

According to Table 3, Model 2 (H2) examined whether difficulties in emotion regulation (DERS) moderate the relationship between digital literacy (DL) and cyberbullying sensibility (CBS). The results indicated that the interaction effect between digital literacy and difficulties in emotion regulation was statistically significant [*b* = −0.015, *p* < 0.001, 95% CI (−0.021, −0.009)].

**Table 3:** Testing the moderating effect of difficulties in emotion regulation.

Predictors model 2 (H2)	CBS (Y)			
	*b*	*t*	SE	95% CI
DL (X)	0.802^***^	4,81	0.166	0.474/1.13
DERS (W)	0.492^***^	3.66	0.134	0.227/0.756
DL × DERS (Interaction)	−0.015^***^	−4.87	0.003	−0.021/−0.009
*R* ^2^	0.124			

Bootstrap sample size = 5,000. LL, low limit; CI, confidence interval; UL, upper limit. ^***^p < 0.001.

In addition, digital literacy significantly predicted cyberbullying sensibility [*b* = 0.802, *p* < 0.001, 95% CI (0.474, 1.130)]. Difficulties in emotion regulation also showed a significant direct relationship with cyberbullying sensibility [*b* = 0.492, *p* < 0.001, 95% CI (0.227, 0.756)].

These findings suggest that the relationship between digital literacy and cyberbullying sensibility varies according to individuals' levels of difficulties in emotion regulation. Specifically, the positive association between digital literacy and cyberbullying sensibility was found to be less pronounced among individuals reporting higher levels of difficulties in emotion regulation, supporting H2.

Model 3 (H3) presented in Table 4 examined whether the indirect relationship between social media addiction (SMA) and cyberbullying sensibility (CBS) through digital literacy (DL) varied according to the level of difficulties in emotion regulation (DERS). The moderated mediation index was found to be statistically significant [*b* = −0.005, 95% CI (−0.010, −0.001)], indicating that the indirect relationship differed across levels of difficulties in emotion regulation.

**Table 4:** Testing the research model.

Predictors model 3 (H3)	CBS (Y)			
	*b*	*t*	SE	95% CI
SMA (X)	0.037	0.624	0.059	−0.080/0.154
DL (M)	0.805^***^	4.82	0.166	0.476/1.13
DERS (W)	0.498^***^	3.69	0.134	0.232/0.764
DL × DERS (Interaction)	−0.015^***^	−4.91	0.003	−0.021/−0.009
*R* ^2^	0.125			
Indirect impact	*b*	SE	95% CI	
Low DERS (*W* = 34)	0.109^*^	0.047	0.025/0.219	
Medium DERS (*W* = 42)	0.062	0.044	−0.002/0.149	
High DERS (*W* = 50)	0.015	0.038	−0.054/0.089	
Moderated mediation index	−0.005^*^	0.002	−0.010/−0.001	

Bootstrap sample size = 5,000. LL, low limit; CI, confidence interval; UL, upper limit. ^***^p < 0.001, ^**^p < 0.01, ^*^p < 0.05.

The indirect effect was strongest at low levels of difficulties in emotion regulation [DERS = 34; indirect effect = 0.109, 95% CI (0.025, 0.219)]. This effect decreased at moderate levels of difficulties in emotion regulation [DERS = 42; indirect effect = 0.062, 95% CI (−0.002, 0.149)] and was not statistically significant at high levels [DERS = 50; indirect effect = 0.015, 95% CI (−0.054, 0.089)].

**Figure 2:**
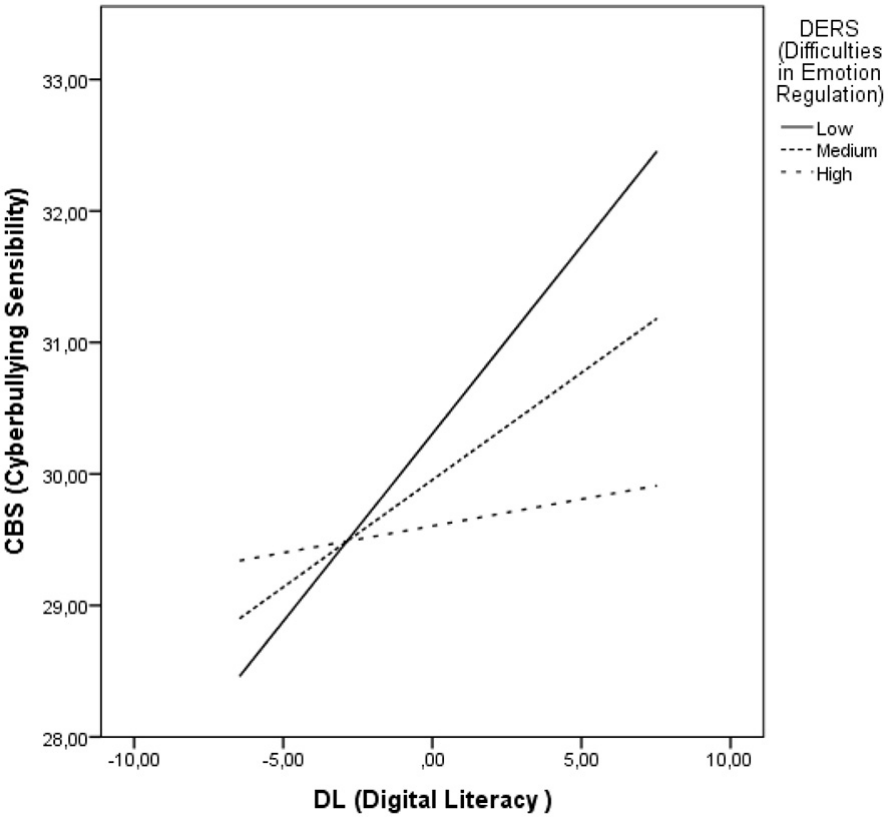
The relationship between digital literacy and cyberbullying sensibility when difficulties in emotion regulation is low, medium and high.

These findings suggest that the indirect relationship between social media addiction and cyberbullying sensibility through digital literacy varies across different levels of difficulties in emotion regulation, supporting H3. The conditional indirect associations at low, moderate, and high levels of difficulties in emotion regulation are illustrated in Figure 2.

## Discussion

The findings of the present study indicate that digital literacy is significantly associated with cyberbullying sensibility among team sports athletes. In addition, difficulties in emotion regulation were found to moderate the indirect association between social media addiction and cyberbullying sensibility through digital literacy. Specifically, the indirect association was observed to be stronger among individuals with lower difficulties in emotion regulation, whereas this association was not statistically significant among individuals with higher difficulties in emotion regulation. These results suggest that cyberbullying sensibility in digital environments may be better understood by considering the interaction of cognitive and emotional factors. Accordingly, the findings provide evidence for a conditional association pattern consistent with the proposed moderated mediation model.

The theoretical basis of the study was grounded in Social Cognitive Theory (Bandura, 1986) and Cognitive Appraisal Theory (Lazarus and Folkman, 1984), both of which emphasize the role of cognitive and emotional processes in shaping individuals' responses to environmental experiences. According to Social Cognitive Theory, behaviors are shaped through the reciprocal interaction of environmental influences, personal characteristics, and prior experiences (Bandura, 1986; Wang et al., 2021). Within digital environments, individuals may internalize behavioral patterns through repeated exposure to online content and social interactions (Stefanone et al., 2011). In this context, excessive engagement with social media may increase exposure to harmful online experiences and influence individuals' psychological responses to digital interactions.

The findings of the present study demonstrated that digital literacy played a significant mediating role in the relationship between social media addiction and cyberbullying sensibility. This finding is consistent with previous research suggesting that online experiences and digital interaction patterns may shape individuals' psychological responses and awareness of online risks. For example, Kara and Aslan (2025) reported that cyberbullying victimization negatively affected self-esteem, whereas social support and self-esteem functioned as protective factors for subjective wellbeing. Similarly, previous studies have shown that cyberbullying experiences in social media environments are associated with psychological outcomes such as stress, anxiety, and depressive symptoms (Kokkinos and Saripanidis, 2017; Lapierre and Dane, 2020). These negative outcomes may become more pronounced in individuals with insufficient digital literacy and limited awareness of online risks (Acar, 2024).

The findings are also consistent with previous research conducted with athletes. (Yücetürk and Agin 2022) emphasized that athletes need greater awareness and education regarding social media-related risks and cyberbullying experiences. In this respect, the present findings suggest that digital literacy may contribute to athletes' awareness of harmful online behaviors and their sensitivity toward cyberbullying in digital environments.

From the perspective of Cognitive Appraisal Theory, individuals' emotional and behavioral responses are shaped by how they cognitively evaluate stressful experiences (Folkman, 2020). Cyberbullying experiences may be perceived as threatening or uncontrollable, and these appraisals may influence emotional reactions and coping processes (Anderson and Hunter, 2012). Previous research has shown that individuals who perceive cyberbullying experiences as less controllable tend to experience higher levels of emotional distress and adopt less effective coping strategies (Völlink et al., 2013). Similarly, Martín-Babarro (2014) suggested that experiences of online exclusion and victimization may lead to intense emotional stress reactions and difficulties in emotional adjustment.

Consistent with this perspective, the findings of the present study indicate that difficulties in emotion regulation weaken the indirect relationship between social media addiction and cyberbullying sensibility through digital literacy. Athletes with lower difficulties in emotion regulation may respond more adaptively to stressful online experiences and may therefore demonstrate greater awareness of cyberbullying-related risks. In contrast, greater difficulties in emotion regulation may increase psychological vulnerability in digital environments and reduce the protective role associated with digital literacy.

## Conclusion

### Theoretical implications

The present study contributes to the literature by examining the relationships between social media addiction, digital literacy, and cyberbullying sensibility within a moderated mediation framework. Consistent with Social Cognitive Theory (Bandura, 1986), the findings suggest that digital literacy may be closely linked to how individuals' awareness and responses in digital environments. In addition, within the framework of Cognitive Appraisal Theory (Lazarus and Folkman, 1984), the findings indicate that difficulties in emotion regulation may influence how individuals respond to harmful online experiences.

In this respect, the study highlights the importance of considering both cognitive and emotional processes together when examining cyberbullying sensibility among athletes. Digital literacy appears to function as an important cognitive resource in recognizing and managing online risks, whereas difficulties in emotion regulation may influence the strength of this relationship.

### Practical implications

The findings suggest that athletes may benefit from educational and psychological support programs aimed at improving digital awareness and coping skills in online environments. In particular, digital literacy and digital ethics-based training programs may help athletes recognize harmful online behaviors and increase awareness regarding cyberbullying risks.

Previous research has also shown that digitally supported intervention programs may contribute to reducing bullying-related experiences and improving awareness in educational settings (Arango et al., 2024). In line with these findings, developing structured educational programs focusing on safe digital interaction and online awareness may be beneficial for athletes and sports communities.

Furthermore, considering the role of difficulties in emotion regulation in digital experiences, psychoeducational programs aimed at strengthening emotional regulation capacities may also help athletes cope more effectively with stressful online interactions. Integrating emotional awareness and coping strategies into digital literacy education may contribute to both individual psychological resilience and healthier online communication environments.

### Limitations and future research

Several limitations should be considered when interpreting the findings of this study. First, the sample consisted only of licensed team sports athletes from Erzincan, Türkiye, which may limit the generalizability of the findings to different cultural or demographic contexts. Future studies may examine the proposed model in different athlete populations and cultural settings.

Second, the study included only team sports athletes. Therefore, the findings may not be directly generalizable to individual athletes, who may experience digital interactions and psychological processes differently. Comparative studies involving both team and individual athletes may provide further insight into these differences.

Third, the cross-sectional design of the study limits the interpretation of causal relationships between variables. Future studies employing longitudinal or experimental designs may provide a more comprehensive understanding of the temporal relationships among social media addiction, digital literacy, emotion regulation, and cyberbullying sensibility.

Finally, the study relied solely on self-report quantitative measures. Since cyberbullying experiences involve complex emotional, cognitive, and social processes, future research using mixed-method or qualitative approaches may provide more detailed and contextualized insights into athletes' digital experiences.

## Data Availability

The raw data supporting the conclusions of this article will be made available by the authors, without undue reservation.
